# Crosstalk of organelles in Parkinson’s disease – MiT family transcription factors as central players in signaling pathways connecting mitochondria and lysosomes

**DOI:** 10.1186/s13024-022-00555-7

**Published:** 2022-07-16

**Authors:** Martin Lang, Peter P. Pramstaller, Irene Pichler

**Affiliations:** 1 Institute for Biomedicine, Eurac Research, Affiliated Institute of the University of Lübeck, Bolzano, Italy; 2 Department of Neurology, University Medical Center Schleswig-Holstein, Campus Lübeck, Lübeck, Germany

**Keywords:** Parkinson’s disease, Lysosome, Autophagy-lysosomal pathway, Mitochondria, MITF, TFEB, TFE3, MiT Transcription factors

## Abstract

Living organisms constantly need to adapt to their surrounding environment and have evolved sophisticated mechanisms to deal with stress. Mitochondria and lysosomes are central organelles in the response to energy and nutrient availability within a cell and act through interconnected mechanisms. However, when such processes become overwhelmed, it can lead to pathologies. Parkinson’s disease (PD) is a common neurodegenerative disorder (NDD) characterized by proteinaceous intracellular inclusions and progressive loss of dopaminergic neurons, which causes motor and non-motor symptoms. Genetic and environmental factors may contribute to the disease etiology. Mitochondrial dysfunction has long been recognized as a hallmark of PD pathogenesis, and several aspects of mitochondrial biology are impaired in PD patients and models. In addition, defects of the autophagy-lysosomal pathway have extensively been observed in cell and animal models as well as PD patients’ brains, where constitutive autophagy is indispensable for adaptation to stress and energy deficiency. Genetic and molecular studies have shown that the functions of mitochondria and lysosomal compartments are tightly linked and influence each other. Connections between these organelles are constituted among others by mitophagy, organellar dynamics and cellular signaling cascades, such as calcium (Ca^2+^) and mTOR (mammalian target of rapamycin) signaling and the activation of transcription factors. Members of the Microphthalmia-associated transcription factor family (MiT), including MITF, TFE3 and TFEB, play a central role in regulating cellular homeostasis in response to metabolic pressure and are considered master regulators of lysosomal biogenesis. As such, they are part of the interconnection between mitochondria and lysosome functions and therefore represent attractive targets for therapeutic approaches against NDD, including PD. The activation of MiT transcription factors through genetic and pharmacological approaches have shown encouraging results at ameliorating PD-related phenotypes in in vitro and in vivo models. In this review, we summarize the relationship between mitochondrial and autophagy-lysosomal functions in the context of PD etiology and focus on the role of the MiT pathway and its potential as pharmacological target against PD.

## Background

The definition of life includes the ability to respond to change. Living organisms constantly interact with their surrounding environment, which requires adaptation to varying conditions. At the systems and cellular level, sophisticated mechanisms have evolved to adapt to changing environments and stress. One such mechanism is lysosomal degradation and macroautophagy, which plays an important role not only in the “garbage disposal” of a cell, but also in metabolic and signaling pathways. Anabolic and catabolic processes of macromolecules and metabolites importantly factor into the adaptation to the environment. In eucaryotes, mitochondria play a central function in this process.

Whenever any of such systems become defective or overwhelmed it can lead to disease. Not surprisingly, mitochondrial and lysosomal functions are implicated in several pathological states, which can be directly related to the organelles, such as mitochondriopathies or lysosomal storage diseases, or more nuanced in an interplay between different organelles, for instance in neurodegenerative diseases (NDDs) or cancer. NDDs, such as Parkinson’s disease (PD) or Alzheimer’s disease (AD), present examples of pathologies where the inability to dispose of proteinaceous aggregates leads to disease. Hallmarks of these NDDs include pathological protein misfolding, aggregation, and accumulation as well as decreased organellar functions that can lead to neuronal dysfunction and cell death, resulting in loss of synaptic connections and brain damage [1, 2].

In an attempt to move from fighting symptoms to tackling the fundamental disease processes in NDDs, current research efforts aim to understand and alter these underlying pathological mechanisms. One pathway that has gained much attention over the last years is the autophagy-lysosomal pathway (ALP), which is involved in maintaining organellar health and clearance of pathological aggregates. Improving the function of this pathway may prove beneficial at maintaining cellular health and therefore slowing down the disease onset or progression [3, 4]. The Microphthalmia-associated transcription factor family (MiT) constitute the master regulators of lysosomal biogenesis [5–7] and as such present plausible targets in the search for disease-altering interventions against NDDs, including PD.

In this review, we summarize the connections between mitochondrial and autophagy-lysosomal functions in the context of PD. The review places a focus on the biology and role of the MiT family of transcription factors at the intersection between mitochondrial and lysosomal functions. We describe the role of the MiT pathway in the context of PD and its potential as pharmacological target against NDDs.

## Main text

### Parkinson’s disease – background and biology

Parkinson’s disease (PD) is the second most common neurodegenerative disorder affecting over 2% of the population over 65 years of age with a particularly high prevalence in Europe and North America that is globally rising [8–10]. The neuropathological hallmarks of PD include the progressive loss of dopaminergic neurons in the *substantia nigra pars compacta* (*SNpc*) and the occurrence of proteinaceous inclusions known as Lewy bodies in the remaining neurons [11, 12]. The subsequent dopamine deficiency in the basal ganglia triggers cellular and synaptic dysfunctions, leading to the classical parkinsonian motor symptoms, which include tremor, rigidity, bradykinesia, and postural instability. In addition, significant non-motor symptoms, such as mental health issues, sleep disorders, pain, or fatigue are associated with PD and can largely precede the motor symptoms [9]. At present, only symptomatic treatments are available for PD, with levodopa (L-Dopa) as the gold standard, and so during treatment the disease pathology continues to progress.

PD has been widely accepted as a multifactorial disorder, with both genetic and environmental factors playing an important role, and age being the biggest risk factor [13]. The large majority of PD cases are classified as idiopathic (iPD, i.e., with an unknown etiology, ~ 90%), and about 10% of cases represent rare monogenic forms with Mendelian inheritance patterns, with more than 20 genes identified to date [14]. However, many of these genes still lack replication and some were not associated with typical PD but rather parkinsonism with or without dystonia. In addition, about 90 common variants, which exert a small increase in PD risk, were identified by genome-wide association studies [15]. Several environmental risk factors, including pesticide exposure and traumatic brain injury, were associated with the risk of developing PD. Conversely, cigarette smoking, caffeine intake and physical activity were described as protective factors [16–18].

Independent of the underlying cause of PD in a single patient, the disease shares similar outcomes and cellular phenotypes. At the cellular and mechanistic levels, such hallmarks of PD are constituted by mitochondrial dysfunction, reactive oxygen species (ROS) and calcium (Ca^2+^) homeostasis, lysosomal and proteasomal dysfunction, and iron and other metal metabolism. These defects can lead to synaptic dysfunction, apoptosis of neuronal cells and neuroinflammation (extensively reviewed by Antony et al., 2013 [19]). The precise disease cascade and correlations between different cellular dysfunctions may be specific to the underlying cause of PD and is still being intensively investigated.

In the pursuit to uncover the biological basis of PD, defects of **mitochondria** and their quality control machinery became evident early on. In the 1980s a link between MPTP (1-methyl-4-phenyl-1,2,3,6-tetrahydropyridine) exposure and parkinsonism was established [20, 21] and attributed to inhibition of respiratory chain complex I by the MPTP derivative MPP^+^ (1-methyl-4-phenylpyridinium) [22, 23]. Exposure to environmental inhibitors of the electron transport chain, such as the pesticide rotenone, has also been associated with an increased risk for PD [24]. The link between mitochondrial malfunction and PD was further corroborated with the description of reduced respiratory chain complex activities in *post-mortem SNpc* tissues as well as platelets, lymphocytes, and fibroblasts of iPD and genetic PD patients [25–29].

Genetic evidence validates the correlation between mitochondrial function and PD etiology, since many familial PD genes either directly (e.g., *PINK1*, *PRKN* (Parkin), *DJ-1*, *CHCHD2*) or indirectly (e.g., *SNCA*, *LRRK2*, *ATP13A2*, *FBXO7*, *VPS13C, VPS35,* and *GBA1*) affect mitochondrial function [30]. Inherited genetic alterations and exposure to environmental toxins can therefore influence multiple aspects of mitochondrial function, including their quality control, dynamics and degradation, intracellular transport as well as metabolic capacity. Further, ageing has been proposed to decrease cellular compensatory mechanisms in post-mitotic tissues [31]. In addition, accumulations of variants in the mitochondrial genome (mtDNA), including point mutations, large deletions, copy number alterations and 7S DNA activity were shown to be affected in various PD patient tissues [32]. Cytoplasmic hybrid (cybrid) cell models have been extensively used to study the function of mitochondria and mtDNA derived from PD patient cells and have mostly corroborated the presence of mitochondrial defects [32]. However, due to large variabilities in this model, some studies could observe only little effect of PD mitochondria on cellular phenotypes or no significant alterations of electron transport chain subunit protein levels [33, 34]. A polygenic enrichment of common genetic variants affecting mitochondrial genes and mtDNA maintenance pathways may further influence the risk of iPD [35, 36]. Mitochondrial dysfunctions can reflect on and impair other cellular functions through multiple ways, including energy and metabolite availability.

Likewise, the involvement of the **autophagy-lysosomal pathway (ALP)** as one of the main cellular mechanisms responsible for effective protein and organelle turnover, has gained strong genetic support for its implication in the pathogenesis of PD. A pathological correlation between lysosomal storage disorders (LSD) and PD has been postulated, and a significant burden of variants in LSD genes was associated with iPD susceptibility [37]. As a prominent example, pathogenic *GBA1* variants, an important PD risk factor, directly affect lysosomal function through reduced lysosomal glucocerebrosidase (GCase) activity [38]. Likewise, alpha-Synuclein (α-Syn) accumulations can lead to impaired autophagy function by affecting autophagosome formation and clearance, and trafficking of lysosomal enzymes [39–41]. Conversely, impaired lysosomal function can exacerbate α-Syn accumulations, as exemplified by mutations in *LRRK2*, which lead to reduced autophagic activity, resulting in α-Syn accumulations [42, 43]. Several additional genes linked to genetic PD or atypical forms of PD are involved in ALP functions, cellular trafficking, endo- and exocytosis, including *ATP13A2*, *PLA2G6*, *ATP6AP2* and two novel PD genes, *VPS13C* and *ATP10B* (reviewed recently by Smolders and Van Broeckhoven, 2020 [44]). Furthermore, ALP functions appear to decrease during ageing (reviewed in [45]).

The principal and most direct interaction between mitochondria and lysosomes is constituted by **mitophagy**, a mechanism of bulk disposal of mitochondrial organelles by the macroautophagy machinery. This highly regulated process is integrated with other mitochondrial quality control systems that include mitochondrial dynamics as well as disposal of single mitochondrial proteins and protein complexes. The canonical mitophagy pathway involves two main proteins, Parkin and PINK1, mutations in which predispose to PD [46–48]. Under normal physiological conditions, PINK1 is recruited to mitochondria and internalized through the outer and inner mitochondrial membrane complexes (TOM and TIM), cleaved by the protease PARL, and readily targeted for degradation by the proteasome [49, 50]. Upon depolarization of mitochondrial membranes, PINK1 accumulates on the outer mitochondrial membrane, where it recruits Parkin and triggers its ubiquitination activity towards other mitochondrial proteins. This leads to a recognition of the ubiquitin-tagged mitochondrial organelle by LC3 cargo receptors, which initiate their degradation by the autophagy machinery [51]. However, while mitophagy can be detected through reporter systems, its role under basal physiological conditions is still debated, and recent data indicate that basal mitophagy in vivo might occur independently of the PINK1-Parkin pathway [52–54]. Examples of Parkin-independent autophagy were reported to involve other E3 ubiquitin ligases and largely follow the PINK1-Parkin downstream pathway for degradation of labeled mitochondria through the autophagy machinery [55].

### Connection between mitochondrial quality control and autophagy-lysosomal pathway in PD

#### Connection between mitochondrial and ALP dysfunctions

Metabolism is an important component of stress response because it can be wired towards anabolism (i.e., creation of building blocks) and catabolism (i.e., conversion of fuel into energy) to respond to the needs of a cell. Mitochondria and lysosomes are central to these metabolic pathways and unsurprisingly, there is a high interdependence of function and malfunction of these organelles. Coordination of organelles can be regulated at various levels, such as biogenesis and removal, organelle positioning within the cell, and regulation of enzymatic activities within the organelles. Furthermore, retrograde signaling from organelles towards the nucleus constitute feedback loops that allow to adapt gene expression signatures to their functional state (reviewed by Deus et al., 2020 [56]).

In addition to the mechanism of mitophagy, direct interactions of mitochondria with lysosomes participate in regulating mitochondrial dynamics and function. In non-neuronal cells, mitochondria and lysosomes form inter-organelle membrane contact sites that are distinct between healthy and damaged mitochondria and mark mitochondrial fission events. Organellar contact tethering is promoted by lysosomal GTP-bound RAB7, and release is driven by mitochondrial TBC1D15-stimulated GTP hydrolysis [57]. In neuronal cell models, mitochondria-lysosomal contacts were shown to form dynamically in multiple cellular compartments under physiological conditions but are influenced by GCase activity. Defects of GCase in patient derived neurons caused a TBC1D15-dependent alteration of mitochondria-lysosome contacts, resulting in disrupted mitochondrial distribution and function [58]. Similarly, mitochondria-endoplasmic reticulum (ER) contact sites may serve as source of membranes for autophagosomes [59].

In relation to organellar dynamics it is of interest to note that mammalian cells have evolved several mitochondria-specific isoforms of membrane-remodeling proteins from the endocytosis machinery (e.g., dynamins, endophilins), which suggests a tight regulation of this pathway. Mitochondrial dynamics proteins and endosomal trafficking proteins, such as VPS35, functionally interact to regulate organellar dynamics [60]. Microtubule function and cytoskeleton organization, which are at the basis of functional organellar dynamics, are influenced by energy availability – and therefore mitochondrial function – and relate back to mitochondrial trafficking defects as well as autophagic defects [61].

Besides physical interactions and indirect functional interactions, signaling pathways connect different cellular organelles. Signaling of mitochondrial dysfunction towards the lysosome has been described in various models. For example, in T-lymphocytes with a knockout of *TFAM* (mitochondrial transcription factor A), which is essential for mtDNA replication and transcription, besides mtDNA depletion and mitochondrial dysfunction also an accumulation of autophagy intermediates was observed, consistent with lysosomal malfunction [62]. Furthermore, enlarged lysosomal vesicles with decreased hydrolytic activity were described in mouse embryonic fibroblasts with a deficiency in OPA1, a mitochondrial fusion factor, or AIF, an apoptosis inducing factor involved in mitochondrial function [63]. Notably, mitochondrial malfunction positively regulates lysosomal biogenesis, which requires MiT transcription factors, particularly TFEB and MITF, and involves AMPK (AMP-dependent protein kinase) and mTORC1 (mammalian target of rapamycin complex 1) regulation. AMPK and mTORC1 signaling pathways constitute major antagonistic metabolic hubs that integrate metabolic signatures. While mTORC1 drives protein synthesis based on metabolic availabilities of the cell, AMPK promotes mitochondrial and lysosomal biogenesis and autophagy under conditions of acute stress (e.g., induced by a mitochondrial toxin) but not under conditions of chronic mitochondrial stress (e.g., a stable knockdown of one of the mitochondrial respiratory chain components) [64, 65]. This might be due to the fact that lysosomal biogenesis is necessary for the degradation of defective mitochondria through mitophagy, but a continuous, chronic elimination of mitochondria cannot be afforded.

On the other hand, also lysosomal stress (e.g., induced by the accumulation of a specific substrate) and the resulting lysosomal dysfunction has an impact on mitochondrial function. The lysosomal storage disorders Niemann-Pick type C (NPC) and acid sphingomyelinase deficiency (ASM) are characterized by the repression of a transcriptional program for mitochondrial biogenesis and function [66]. Autosomal recessive Gaucher’s disease (GD), the most common LSD, is caused by bi-allelic loss of function mutations of *GBA1* and is characterized by glucosylceramide accumulation in various organs and cells and can involve neurodegenerative features [67]. The *GBA1* protein product GCase is a lysosomal enzyme responsible for the hydrolysis of the glycosphingolipid glucocerebroside to ceramide and glucose. In GD, loss of GCase activity leads to accumulation of sphingolipids, including glucocerebroside, in the lysosomes. This can alter membrane fluidity [68] and lead to an increase in lysosomal pH and lysosomal destabilization and hence affect autophagic cargo degradation. The accumulation of mutant GCase protein may further lead to ER stress, resulting in unfolded protein response and related cellular phenotypes, including apoptosis [69].

In addition to lysosomal defects, GD models have demonstrated that lysosomal and autophagy defects can result in downstream accumulation of dysfunctional mitochondria. Inhibition of GCase in cell lines leads to oxidative stress and mitochondrial dysfunction, including decreased mitochondrial membrane potential and ATP production, and increased ROS formation ([70], reviewed in [71]). Mitochondrial dysfunction was also shown in *GBA1* mutated fibroblasts from GD patients [70]. Furthermore, *Gba1* knockout as well as *Gba1* gain of function mutations in mouse models resulted in increased mitochondrial fragmentation, reduced oxygen consumption, oxidative phosphorylation activity and ATP production [72, 73].

Lysosomal storage disease models also evidenced that perturbation of Ca^2+^ homeostasis can be an important factor in linking mitochondrial and lysosomal functions. Ca^2+^ is an important second messenger that regulates numerous cellular processes, including Ca^2+^-dependent effector proteins (e.g., kinases, phosphatases, ion channels) and Ca^2+^-dependent functions (e.g., metabolism, apoptosis, lysosomal function). The ER is the largest Ca^2+^ store in the cell and close contacts between ER and other organelles, such as mitochondria and lysosomes, help regulate and fine-tune Ca^2+^ homeostasis [74]. Contact sites between mitochondria and lysosomes were found to modulate mitochondrial dynamics through modulation of GTPase activities [57], and Ca^2+^ transport at mitochondria–lysosome contact sites is mediated by the non-selective lysosomal cation channel MCOLN1 (mucolipin1, also TRPML1), which can regulate autophagy through Ca^2+^ release from the lysosomes [75]. Ca^2+^- and calcineurin-driven activation of TFEB is central to this mechanism [76]. Thereby, Ca^2+^ metabolism may play a role in the pathophysiology of disorders characterized by dysfunctional mitochondria or lysosomes.

**Fig. 1:**
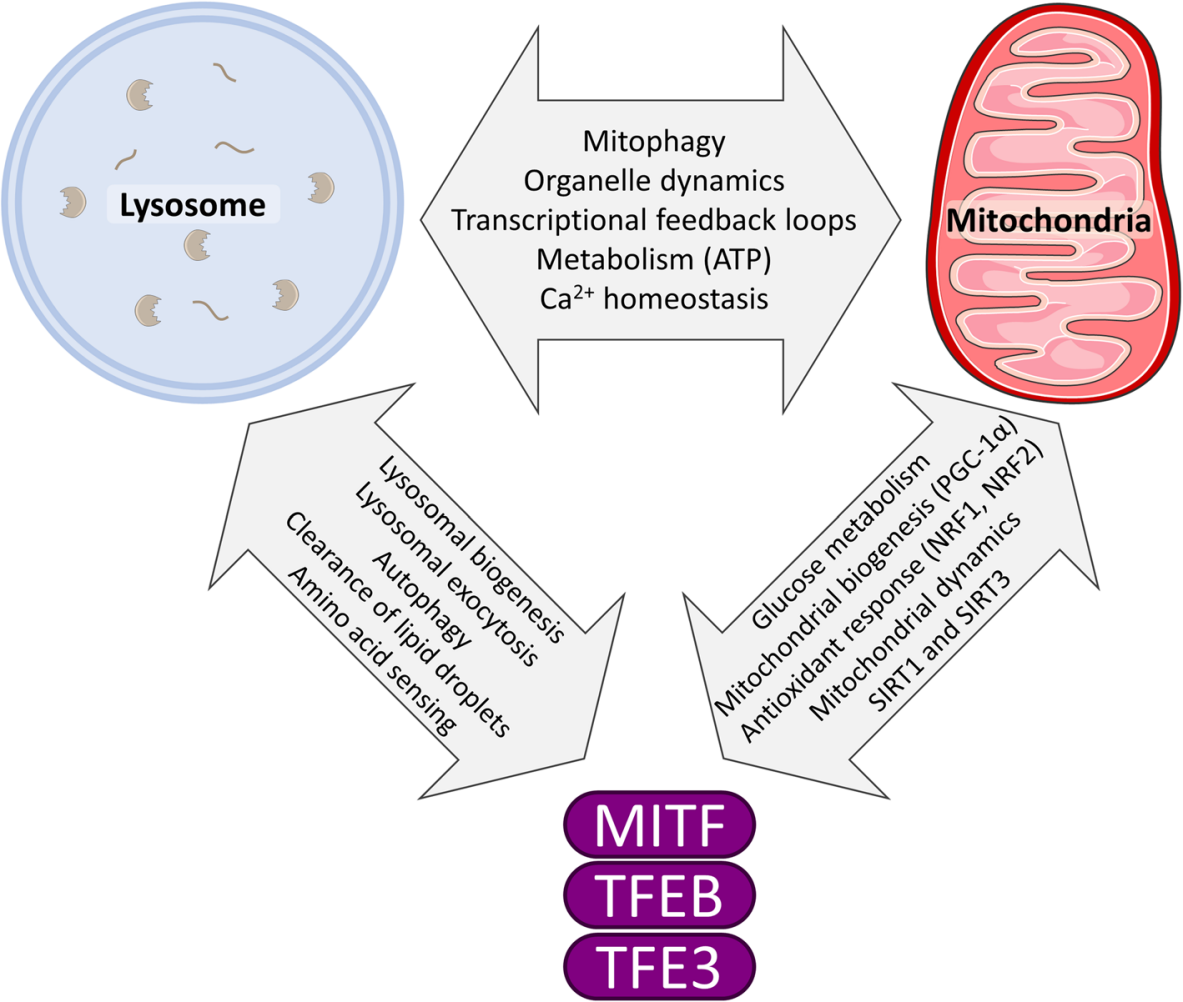
Functional connections between lysosomes, mitochondria and MiT transcription factors. Biological mechanisms linking each organellar function with each other and with MiT transcription factors are schematically outlined. Details on study models and manipulations used to draw conclusions on the mitochondria-lysosomal connection are listed in Table 1. This figure was created using elements from Servier Medical Art, which is licensed under a Creative Commons Attribution 3.0 Unported Generic License (https://creativecommons.org/licenses/by/3.0/)

Lysosomal acidification by v-ATPases requires ATP and therefore senses the energy status of the cell. Consequently, dysfunctional mitochondria resulting in decreased ATP levels impact proper lysosomal acidification and function. Likewise, other lysosomal transporters, such as amino acid transporters, are equally involved in linking the metabolic status, as well as nutrient and metabolite availability of a cell to signaling outputs. Besides their intrinsic physiological function, lysosomes and mitochondria also play a physical role as signaling platforms and hubs for intracellular signal transmission. A prominent example is given by the mTOR complex, whose activity is at least partially linked to its lysosomal localization. This physiological crosstalk between organelles may depend on the context of the study models, tissues, cells, and experimental conditions and should be interpreted accordingly. Table 1 provides a list of references describing the outlined aspects of the mitochondria-lysosome crosstalk and highlights the study models as well as genetic and chemical alterations used to draw conclusions. In addition, functional connections between mitochondria and lysosomes are schematically outlined in Fig. 1.

**Table 1:** Summary of descriptions of mitochondria-lysosome crosstalk

Topic	Conclusion	Cell Type/ model	Genetic/ chemical alterations	Ref
Mitophagy	Parkin is selectively recruited to dysfunctional mitochondria with low membrane potential and promotes autophagy of damaged mitochondria	HEK293; HeLa cells; rat cortical neurons; MEFs	overexpression of fluorescently labeled Parkin; *Mfn* knockouts; CCCP treatment	[47]
Mitophagy	PINK1 signals mitochondrial dysfunction to Parkin, which promotes their degradation	HeLa cells; rat cortical neuron; MEFs	overexpression of fluorescently labeled Parkin and PINK1; *PINK1* mutants and knockout; CCCP and rapalog treatment	[48]
Mitophagy	Parkin amplifies PINK1-mediated mitophagy signals to engage specific autophagy receptors	HEK293T; HeLa cells; rat cortical neurons; MEFs	knockout of autophagy receptor genes; overexpression of fluorescently labeled Parkin and PINK1	[51]
Organelle dynamics	Mitochondrial and lysosomal dynamics is regulated bidirectionally at mitochondria-lysosome contact sites	HeLa cells	overexpression of wt and mutant Rab7-GFP	[57]
Organelle dynamics	Mitochondria-lysosome contacts dynamically form in different neuronal cell compartments and participate in organelle regulation	iPSC-derived neurons with *GBA1* mutation	*TBC1D15* overexpression; GCase inhibition with conduritol-β-epoxide (CBE)	[58]
Organelle dynamics	Autophagosomes originate from ER-mitochondria contact sites	COS7; HeLa; HEK293 cells;	starvation, *STX17* knockdown	[59]
Organelle dynamics	Mitochondrial homeostasis is regulated by the endosomal protein sorting machinery	RPE; HeLa cells	*EHD1*, *Rank5* siRNA; staurosporine; GST–EHD1 expression	[60]
Mitochondria-lysosome crosstalk	Inhibition of GCase activity induces defects in mitochondrial function and oxidative stress in vitro	SH-SY5Y cells	long-term CBE treatment; *GBA1* knockdown	[70]
Mitochondria-lysosome crosstalk	A primary lysosomal defect due to *GBA1* mutations causes accumulation of dysfunctional mitochondria due to impaired autophagy and dysfunctional proteasomal pathways	mouse model of brain Gba1 deficiency; primary neurons	*Gba1* het/hom knockout; LC3-GFP; mitochondrial stressors	[72]
Mitochondria-lysosome crosstalk	GCase deficiency leads to aggregation of multiple proteins and abnormal mitochondrial function in vivo	Gaucher Disease mouse model; cortical neural cells	*Gba1* mutations + hypomorphic prosaposin mutation; CBE treatment	[73]
Transcriptional feedback loop	Mitochondrial regulation of lysosomes is time- and context dependent	MEFs; human fibroblasts; SK-N-MC cells	Mutations in CI genes; CCCP, rotenone treatment	[64]
Transcriptional feedback loop	AMPK plays a central role in mitochondria-lysosomal crosstalk	HeLa cells; MEFs	*UQCRC1*, *FLCN* knockdown; *Ndufs4*, *Prkaa1/2* knockout	[65]
Transcriptional feedback loop	Mitochondrial function is impaired in lysosomal storage disease models	patient fibroblasts; mouse tissues	*NPC1*- and *ASM1*-deficiency; *ETV1*, *KLF2* knockdown	[66]
Transcriptional feedback loop	FLCN is a regulator of AMPK and contributes to the integration of energy metabolism and autophagy	*C. elegans;* MEFs	*flcn-1*, *aak-2* knockdown and mutations; *Ampk*, *Flcn* knockout; cellular stressors	[77]
Metabolism	Dysfunctional mitochondria affect microtubule trafficking and lead to defective autophagy in PD	mtDNA-less Rho0 cells; Cybrid cells from Ctrls and PD patients; primary cortical neurons	Serum, pyruvate/uridine starvation, lysosomal proteolysis inhibition; MPP +	[61]
Metabolism	Impaired mitochondrial metabolism affects endolysosomal function in T-cells	mouse T-cells; T-lymphoblasts; Jurkat T cells	*Tfam* knockdown and knockout; nicotinamide precursor NAM treatment	[62]
Metabolism	Loss of mitochondrial function impairs lysosomal activity in a ROS-dependent manner	mouse cortical neurons; MEFs	knockout of *AIF*, *OPA1*, *PINK1*; OXPHOS complex inhibitors, antioxidant treatment	[63]
Ca^2+^ homeostasis	Similar to mitochondria, lysosomes can selectively accumulate Ca^2+^ and shape intracellular Ca^2+^ signaling	HEK and COS-7 cells	chemical and genetic disruption of lysosomal function	[74]
Ca^2+^ homeostasis	Mitochondria-lysosome contact sites regulate mitochondrial Ca^2+^ dynamics	HeLa, HEK293, HCT116 cells; fibroblasts	TRPML1 agonist ML-SA1 treatment; *TRPML1* mutant expression	[75]
Ca^2+^ homeostasis	Lysosomal biogenesis and autophagy are regulated through TFEB in a Ca^2+^/MCOLN1-dependent manner	HeLa cells	*PPP3CB* (calcineurin subunit) knockdown and overexpression; TFEB-GFP/TFEB-Flag overexpression; starvation	[76]

A list of studies describing the mitochondria-lysosome crosstalk are summarized to reflect the major functional connections between mitochondria and lysosomes outlined in Fig. 1. Main conclusions, study models and genetic manipulations or chemical treatments used to draw conclusions are shown

#### Connection between mitochondrial and ALP dysfunctions in PD

PD is perhaps the most prominent disease with a well-established link between mitochondrial and ALP dysfunctions. In human PD patient-derived dopaminergic neuron models, a time-dependent pathological cascade was proposed for the link between mitochondria and lysosomes. In this model, mitochondrial oxidant stress led to oxidized dopamine accumulation and ultimately resulted in reduced GCase enzymatic activity, lysosomal dysfunction, and α-Syn accumulation. Here, dopamine oxidation represented an important link between mitochondrial and lysosomal dysfunction, highlighting the need for early therapeutic intervention against PD [78]. In fibroblast models from PD patients, it was shown that mitochondrial and lysosomal functions are synergistically altered, whereby mitochondrial genetic defects might play a role in inducing mitochondrial dysfunction and cellular senescence [79].

**Fig. 2:**
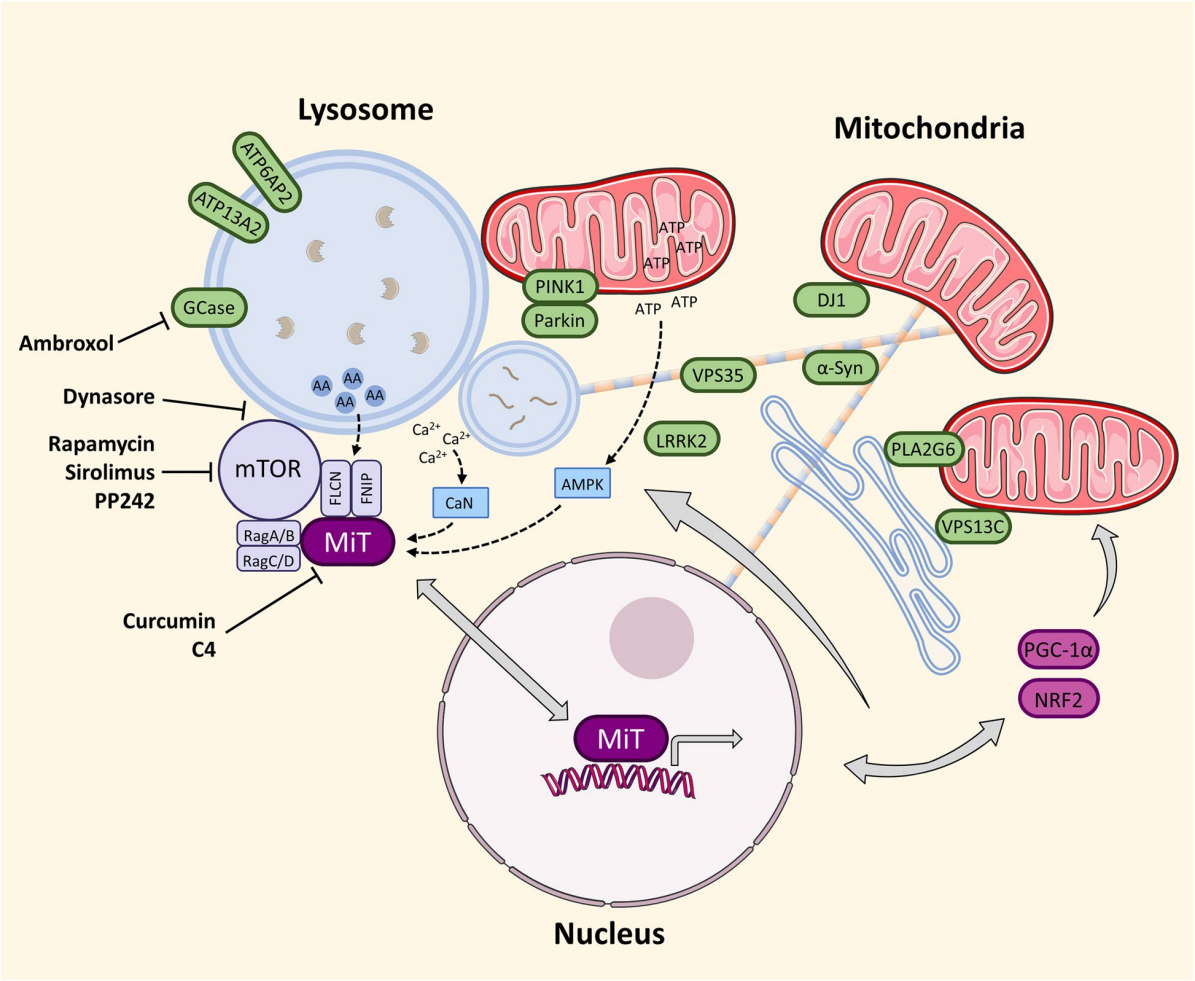
MiT pathway at the intersection between lysosomes and mitochondria in the context of PD-related genes. MiT transcription factors (MITF, TFE3, TFEB) are activated through the mTOR complex in concert with RagA/B, RagC/D and FLCN/FNIP actions in response to amino acid (AA) availability. Upon dephosphorylation and nuclear translocation, MiT proteins activate transcription of genes involved in lysosomal biogenesis and mitochondrial homeostasis, including PGC-1α and NRF2. Proteins marked in green are examples of proteins implicated in familial forms of PD or carry PD risk alleles that are primarily affecting mitochondria or lysosomal functions. Secondary effects of mutations in those genes impair multiple cellular organelles, including membrane homeostasis and cytoskeleton organization, which reflects on mitochondrial dynamics and autophagy. Metabolites, such as ATP and Ca^2+^ connect mitochondrial and lysosomal functions by indirectly affecting the activation of MiT members through AMPK and Calcineurin (CaN), respectively. Small molecule inhibitors affecting MiT activation are indicated with blunt arrows pointing towards their protein targets. This figure was created using elements from Servier Medical Art, which is licensed under a Creative Commons Attribution 3.0 Unported Generic License (https://creativecommons.org/licenses/by/3.0/)

The crosstalk between mitochondrial and lysosomal function becomes particularly evident from numerous genes implicated in familial forms of PD and PD risk alleles. Strikingly, many genes that are directly involved in autophagic and endo-lysosomal pathways also induce mitochondrial defects when mutated, while mitochondrial dysfunction due to mutations in mitochondrial genes can impact lysosomal function. Prominent examples of such genes are listed here and schematically placed within the cellular context in Fig. 2.

- **PINK1** (PTEN Induced Kinase 1) and **Parkin** (*PRKN*) are central players of the classical form of mitophagy and thus directly mediate mitochondrial quality control by targeting dysfunctional organelles towards degradation by the autophagy machinery [46, 80].
- **DJ-1** (*PARK7*) is a mitochondrial redox-sensitive chaperone that functionally interacts with PINK1 [81]. Mutations in this gene can affect mitochondrial and autophagy homeostasis [82, 83].
- **Alpha-Synuclein** (α-Syn) is involved in synaptic vesicle trafficking [84]. Gain of function mutations can lead to α-Syn accumulation, oligomerization into fibrils and Lewy body formation, which induces autophagy and mitochondrial impairments [85].
- The GTPase ***LRRK2*** (Leucine-Rich Repeat Kinase 2), which is mutated in ~ 40% of all genetic forms of PD, interacts with many autophagosomal and lysosomal proteins, but was also shown to impact mitochondrial dynamics and function [86, 87].
- Vacuolar protein sorting-associated protein 35 (**VPS35**) is involved in endosomal trafficking, including mitochondria-derived vesicles [88, 89], and PD-associated mutations in this gene affect mitochondrial dynamics and turnover [90].
- ***ATP13A2*** (ATPase Cation Transporting 13A2), whose mutations can cause a genetic syndrome that involves PD-like symptoms, is a lysosomal ATPase involved in cation homeostasis with the outcome of affecting mitochondrial functions [91, 92].
- Mutations in ***ATP10B*** (ATPase Phospholipid Transporting 10B) have recently been implicated as potential risk factor for PD [93], even though these results could not be confirmed in several large cohorts [94–96]. However, like ATP13A2, ATP10B is a late end-lysosomal ATPase that functions as lipid flippase for glucosylceramide and phosphatidylcholine and may provide protection to cells from environmental risk factors, such as rotenone [93].
- **ATP6AP2** (ATPase H^+^ Transporting Accessory Protein 2), an accessory protein to a vacuolar H^+^-transporting ATPase is involved in lysosomal pH regulation, which is linked to autophagy and possibly mitochondrial functions [97, 98].
- ***PLA2G6*** (Phospholipase A2 Group VI), mutations of which are responsible for autosomal recessive early-onset PD [99], is a Ca^2+^-independent phospholipase, which participates in cell membrane homeostasis, Ca^2+^ signaling, as well as mitochondrial and lysosomal functions [100, 101].
- ***TMEM175*** (Transmembrane Protein 175), another PD risk factor gene, is a lysosomal K^+^ channel involved in maintaining lysosomal pH and, consequently, mitochondrial health [102].
- **SREBF1** (Sterol Regulatory Element Binding Transcription Factor 1) is a transcription factor that plays a key role in lipogenesis but has also been implicated in Parkin-mediated mitophagy [103].
- Mutations in vacuolar protein sorting 13C (***VPS13C***) cause a severe autosomal-recessive form of parkinsonism. VPS13C was shown to partially localize to the outer mitochondrial membrane as well as endosomes and lysosomes, while mutations affect protein expression and mitochondrial function [104, 105].

Heterozygous mutations of *GBA1,* which encodes lysosomal GCase, are now recognized as the most common risk factors for PD. After initial incidental descriptions of GD patients with parkinsonism, a higher incidence of PD among *GBA1* mutation carriers was found; *GBA1* mutations confer a 20–30-fold increased risk to develop PD, and overall, 5–10% of PD patients have been found to carry *GBA1* variants [106, 107].

Cell, animal, and patient studies with *GBA1* mutations have shown autophagy defects that lead to mitochondrial dysfunction and can involve α-Syn pathology [108]. The exact mechanism, however, is not clear yet. *Drosophila* studies of GCase deficiency were able to model neurodegeneration phenotypes and accelerated protein aggregations. A recent study presented evidence that dysregulation of extracellular vesicles contributes to the spread of protein aggregates between cells and tissues in the context of GCase deficiency [109]. Furthermore, GCase deficiency exacerbated neurotoxicity and neurological phenotypes of α-Syn overexpression in flies due to an aberrant interaction between α-Syn and glycolipids [110]. However, the role of sphingolipids in this context in human PD is still debated, since its accumulation mainly happens under *GBA1* knockout conditions, which is often not observed in PD. Discrepant results on accumulation of glucosylsphingosine have been reported in different brain regions of PD patients [111, 112]. On the other hand, direct cross-functional interactions between GCase and α-Syn may explain some of the GCase-deficiency phenotype, as accumulation of mutated GCase or reduced lysosomal-mediated autophagy can lead to inhibition of α-Syn degradation [113]. Furthermore, *GBA1* mutations have been shown to lead to complex changes in ALP and intracellular Ca^2+^ homeostasis in induced pluripotent stem cell (iPSC)-derived neuron models, which can add to cellular vulnerabilities in NDDs [114]. Also, direct physical contacts between mitochondria and lysosomes were shown to be prolonged in PD patient-derived neurons with *GBA1* mutations leading to disrupted mitochondrial distribution and function [58].

In summary, PD causative and risk factor genes directly affect mitochondrial or lysosomal pathways with frequent functional consequences to the other organelle compartment. While both organelles will be affected in the full disease state, the pathogenic cascade of disease progression is still being studied. For the development of early therapeutic interventions against PD, it will be important to shed light on the flow of events. Different genetic backgrounds may present specific disease progression pathways, and personalized medicine approaches are warranted to distinguish molecular subtypes for treatment purposes.

### MiT family of transcription factors – master regulators of lysosomal biogenesis

Mitochondria play a central role in the metabolism of the cell, principally through the citric acid cycle and oxidative phosphorylation, in addition to numerous connected biosynthetic pathways, such as nucleotide, fatty acid, and amino acid biosynthesis. Macroautophagy and lysosomal degradation of defective organelles, cellular debris and protein aggregates are at the basis of keeping a clean cellular household. This regulated mechanism should be understood as much more than a “garbage disposal” of the cell and stands at the basis of cellular homeostasis and cellular health. The differentiated role of macroautophagy in metabolism and organellar quality control has recently been comprehensively reviewed [115]. Over the last few years, basic research efforts have shed light on the mechanisms behind the interplay between PD-related mitochondrial defects, lysosomal dysfunctions, and protein aggregate formations in the context of PD. One of the central hubs regulating cellular metabolism and homeostasis is the AMPK and mTOR signaling pathway that links mitochondrial and lysosomal functions. A fringe player in this pathway, folliculin (FLCN), provides the rheostat for amino-acid sensing and lysosomal biogenesis (reviewed in [116]). Regulation of the mTOR pathway via FLCN can trigger a downstream transcriptional program aimed at responding to nutrient stress through the activation of the MIT family of transcription factors, to stimulate lysosomal biogenesis and autophagy.

### Overview of MiT family of transcription factors

The Microphthalmia-associated transcription factor family includes the basic helix-loop-helix domain-containing transcription factors MITF, TFE3, TFEB, and TFEC. Members of this transcription factor family share similar protein structures, recognize and bind E-box DNA sequences upon homo- or heterodimerization among each other, and promote the transcription of similar genes [117, 118]. Subcellular localization of MiT transcription factors is tightly regulated through post-translational modifications and is crucial for their activation (extensively reviewed by Puertollano et al., 2018 [119]). MiT transcription factors are considered the master regulators of autophagy and lysosomal biogenesis [5–7, 120] and have been shown to play a central role in regulating cellular homeostasis in response to nutrient availability [120–123], exit from pluripotency during stem cell differentiation [124, 125], and melanocyte development [126].

#### MITF

Microphthalmia-Associated Transcription Factor (MITF) regulates a transcriptional program involved in the development and differentiation of melanocytes, osteoclasts, and mast cells. MITF also regulates pigment cell-specific transcription of melanogenesis enzyme genes. Accordingly, germline mutations in *MITF* are responsible for the autosomal dominant Waardenburg syndrome type II and the more severe and rare Tietz syndrome, both characterized by hearing loss and hypopigmentation of skin, hair, and eyes (OMIM 156845). A common germline variant of *MITF* (p.E318K) has been shown to constitute a risk factor for the development of melanoma and different subtypes of renal cell carcinoma (RCC) by impairing protein sumoylation, which affects its transcriptional activity [127–129]. Somatically, *MITF* gene fusions have been reported in RCC [130, 131], while *MITF* amplification is common in melanoma [132, 133] and aberrant *MITF* transcription is responsible for the oncogenic transformation of clear cell sarcoma subtypes [134].

#### TFE3

TFE3 has mainly been studied in the context of its role as sporadically mutated oncogene in kidneys and lungs, whereby chromosomal translocations underly the formation of fusion proteins involving TFE3 [135]. All TFE3 fusion isoforms retain the wild type C-terminus part of the protein with the DNA binding and dimerization domains, while the fusion partners cause the constitutive nuclear localization of the TFE3 fusion protein, leading to dysregulated transcriptional activity that promotes carcinogenesis [136]. Recently, three reports described patients with point mutations in the *TFE3* gene that led to an X-linked genetic syndrome, characterized by pigmentary mosaicism, intellectual disability, and facial dysmorphisms. The described gain of function mutations showed evidence of mosaicism in some patients and clustered in a region of the protein thought to be involved in Rag-binding and lysosomal-dependent inactivation of the transcription factor [137–139]. These works underline the physiological relevance of this transcription factor in lysosomal biogenesis and development.

#### TFEB

The largest body of work on the biology of MiT transcription factors has so far been performed on TFEB, and its role in regulating the ALP system has been extensively described over the last decade. Similar to TFE3, TFEB is clinically relevant mainly as sporadically mutated oncogene in RCC, where it is frequently involved in a chromosomal translocation with the non-transcribed Alpha gene (*MALAT1*), namely t(6;11), which provides a constitutively active promoter upstream of the *TFEB* gene that drives its transcription [136, 140]. Canonical *TFEB* fusion genes and *TFEB* amplifications have recently been recognized in RCC [130, 141, 142]. These subtle genetic differences between TFE3 and TFEB as tumor drivers may hint towards some specialized cell-type specific and not entirely overlapping mechanisms of regulation of these two transcription factors.

#### TFEC

TFEC variants have so far not clearly been associated with disease. This member of the MiT transcription factor family shows a cell-type specific expression and plays a role in regulating neural crest cells and driving pigment cell development [143]. Further, it is expressed in stimulated bone marrow-derived macrophages [144] and facilitates hematopoiesis [145]. A mechanistic role of TFEC has been described in conditions of cardiac hypertrophy through inhibition of AMPK/mTOR signaling [146].

### Biological function of MiT transcription factors

Transcriptional activity of MiT proteins has been implicated in several signaling pathways affecting cell proliferation and differentiation, regulation of cell cycle and cell–cell interaction (reviewed in [136] and [147]). Most mechanistic studies on this transcription factor family have focused on TFEB and TFE3, which play a major role in the regulation of lysosomal biogenesis and autophagy, thus shaping the cells metabolism [7, 120–123]. MiT members act by binding to a 10-bp DNA motif termed as Coordinated Lysosomal Expression and Regulation (CLEAR) element and activate transcription of downstream genes [5]. TFEB enhances degradation of autophagic substrates [120], lysosomal exocytosis [148], clearance of lipid droplets [149] and mitochondria [150]. Furthermore, genes involved in glucose and lipid metabolism as well as mitochondrial biogenesis are also regulated by CLEAR elements ascribing MiT proteins a role in coordinating oxidative metabolism [149, 151–153] (Fig. 1).

MiT transcription factor activity is mainly regulated at the post-translational level. However, also their transcriptional control is tightly regulated and dependent on cell type and metabolic requirements. Accordingly, TFEB transcription is stimulated by Peroxisome proliferator-activated receptor gamma coactivator-1alpha (*PPARGC1A*, PGC-1α), complexed with retinoid X receptor-alpha (RXRα) and peroxisome proliferator-activated receptor-alpha (PPARα) [149, 154, 155] in neurodegenerative models and under starvation conditions. Upon fasting, CREB (cAMP-response element binding protein) regulates TFEB expression in concert with its coactivator CRTC2 (CREB-regulated transcription coactivator 2) in the liver of animal models [156]. In addition, by activating an autoregulatory feedback loop upon starvation, TFEB can regulate its own transcription [149].

Under physiological circumstances, the posttranslational activation of MiT transcription factors is tightly regulated and linked to the metabolic status of the cell. In the presence of nutrients, TFE3 and TFEB are recruited to lysosomes, where they are phosphorylated by active mTORC1 [157]. Several serine/threonine protein kinases such as Akt, GSK3β, and MAP4K3 [158], as well as protein kinase Cβ (PKCβ) [159] and Src kinase [160] have been shown to phosphorylate TFEB at various residues in addition to – and partially independent of – mTORC1 action. MiT phosphorylation creates a binding site for 14–3–3 chaperones leading to sequestration of the transcription factors in the cytoplasm and their inactivation. Conversely, amino acid deprivation and mTORC1 inactivation allows dephosphorylation and nuclear translocation of the transcription factors, which leads to the upregulation of transcriptional profiles intended to restore nutrient availability of the cell [120–123, 161].

The molecular process of MiT regulation is tightly linked to action of mTORC1. Main properties of the mTOR signaling pathway are outlined in Table 2. MiT transcription factor phosphorylation by mTORC1 is mediated by Rag GTPases via a substrate-specific mechanism that is dependent on the amino acid-mediated activation of RagC and RagD GTPases [162]. Folliculin, encoded by the tumor suppressor gene *FLCN*, is associated with the Birt-Hogg-Dubé syndrome, characterized by fibrofolliculomas, spontaneous pneumothorax and kidney tumors, and its binding partners FNIP1/FNIP2 (FLCN interacting proteins) are involved in Rag-mediated amino acid signaling through mTORC1 and play a role in tuning the conversion between active and inactive mTORC1. A heteroduplex protein complex of FLCN and FNIP1/FNIP2 has GTPase activating protein (GAP) activity towards RagC/D in an amino-acid sensitive fashion, thereby generating GDP-bound RagC/D, which is necessary for mTORC1 activation [163, 164]. In parallel, the guanine nucleotide exchange factor (GEF) activity of Ragulator toward RagA/B results in GTP-loaded RagA/B and recruitment of mTORC1 to the lysosome [162]. This mechanism represents a substrate-specific use of the mTORC1 platform for the regulation of particular cellular nutrients, such as amino acids. While cytoplasmic/nuclear shuttling of MiT proteins is controlled by mTORC1 phosphorylation events, their transcriptional activity was recently shown to be further regulated by AMPK via phosphorylation of specific serine residues [165, 166]. Although most models suggest that FLCN acts as a negative regulator of AMPK, some discrepant results evidence the complex and context-dependent nature of this pathway (reviewed in [116]).

**Table 2:** The mTOR signaling pathway

The **mTOR ** signaling pathway integrates both intracellular and extracellular signals to regulate cell growth, proliferation, differentiation, and survival, and therefore plays an important role in several physiological and pathological settings. mTOR constitutes the basis for the formation of multi-protein complexes (mTORC1, mTORC2) that serve as crucial intermediaries to adapt physiological mechanisms to the cellular metabolic status (comprehensively reviewed in [167]). Because of the fateful downstream effects enacted through mTORC1 activation, this requires both exogenous stimulations and an abundant intracellular nutrient supply. Such examples of centrally integrated pathways are growth factors, glucose, and amino acid signaling pathways.
**Growth factors** activate receptor tyrosine kinases or G-protein-coupled receptors that activate PI3K/Akt (Phosphoinositide 3-kinases/ Protein kinase B (PKB), also known as Akt), which in turn phosphorylate and inhibit TSC (tuberous sclerosis complex), thereby relieving the inhibition of the small GTPase Rheb and allowing it to become activated and stimulate mTORC1 kinase activity.
The cellular **glucose status** is mainly sensed indirectly through AMP/ATP ratio by AMP-activated protein kinase (AMPK), which can act directly, but antagonistically, on mTORC1, as well as in a parallel signaling cascade to regulate glycolysis and mitochondrial biogenesis.
**Amino acids** can signal to mTORC1 through Rag GTPases by different mechanisms, including cytoplasmic sensors, such as GATORs (protein complexes that regulate the activity of RagB), amino acid transporters and the v-ATPase on the lysosomal membrane. For its activation, mTORC1 is recruited to the lysosomal surface via Ras-related small GTPases (Rag) and is allosterically activated by Rheb, a Ras homolog, in its GTP-bound, activated state
**Rags are GTPases** that recruit mTORC1 to the lysosomal surface. They function as obligate heterodimers consisting of RagA or RagB bound to RagC or RagD. The guanine-nucleotide-binding state of the RagA/B–RagC/D heterodimer governs mTORC1 binding, whereby in their active conformation, RagA/B is bound to GTP and RagC/D is bound to GDP. The GTP/GDP binding state of Rags is regulated by amino acid sensors, including FLCN (folliculin) [163, 164, 167]

Accordingly, TFEB is part of the integrated stress response of a cell through its ability to regulate the cell’s response to starvation that requires coordinated decrease of protein synthesis and increased catabolism. Through its activation by inactive mTORC1 and direct stimulation of the eIF2alpha activator GADD34, TFEB adjusts translation events during starvation, thus enabling lysosomal biogenesis and a sustained autophagic flux [168]. A schematic representation of the MiT transcription factor activation pathway at the intersection between lysosomes and mitochondria is shown in Fig. 2.

The activity of MiT transcription factors is further fine-tuned by the regulation of their nucleo-cytoplasmic shuttling through dephosphorylation. TFEB has been shown to continuously shuttle between the cytosol and the nucleus, allowing for additional control over its activity through the regulation of nuclear export. Accordingly, TFEB’s phosphorylation status modulates its nuclear export via CRM1 (Exportin 1) through activation of a nuclear export signal [169, 170]. In addition, Calcineurin and PP2A (Protein Phosphatase 2) have been described as MiT phosphatases that react to changes in intracellular Ca^2+^ levels and acute oxidative stress, respectively, to dephosphorylate and activate MiT proteins, and hence autophagy and lysosomal biogenesis [76, 171].

To further regulate the activity of MiT transcription factors, the transcriptional repressor ZKSCAN3 has been proposed to counteract the activity of TFEB by repressing the expression of an overlapping set of autophagy- and lysosomal genes [172]. However, the role of ZKSCAN3 in vivo as transcriptional repressor of autophagy genes was not supported by a knockout mouse model, raising the question of its importance in a physiological context [173].

### Role of TFEB in mitochondrial quality control

The induction of general autophagy can promote mitophagy [174], and recent evidence link the MiT pathway to an integrated response for the regulation of mitochondrial quality control. The main player connecting the MiT transcription factor cascade with mitochondria is the master regulator of mitochondrial biogenesis PGC-1α. Overlapping upstream pathways can stimulate both, MiT members and PGC-1α. For instance, the two metabolic sensors AMPK and Sirtuin-1 (SIRT1) have both been implicated in regulation of PGC-1α and TFEB. Different in vivo and in vitro models have shown an interdependence of these two metabolic regulators ([154] and reviewed by Canto et al., 2009 [175]). In cellular and animal models of PD with mitochondrial defects, alterations in SIRT1 phosphorylation, decreased mitochondrial Sirtuin-3 (SIRT3) levels and reduced PGC-1α levels have been described. Such alterations could be counteracted by AMPK agonist AICAR [176, 177].

In vitro and in vivo experiments involving overexpression of TFEB in various cell lines and tissues have shown that TFEB can bind to the *PPARGC1A* promoter and directly activate its transcription [149, 152, 153, 178]. Also MITF levels correlated with PGC-1α expression [151]. Moreover, the reverse control seems to be true, wherein PGC-1α can control the activation of TFEB. At least in an in vivo model of Huntington’s disease (HD), PGC-1α overexpression ameliorated the neurodegeneration phenotype through TFEB activation [154]. While TFEB nuclear localization and transcriptional activity were increased upon PGC-1α overexpression [179], PGC-1α knock-out can decrease TFEB and TFE3 expression in skeletal muscle [152, 153]. Together, these data suggest a coordinated mechanism of lysosomal and mitochondrial biogenesis.

In addition to PGC-1α, TFEB has been shown to directly regulate several mitochondrial genes. In a mouse model of muscle-specific gain and loss of function study for TFEB during physical activity, TFEB was shown to regulate glucose uptake and glycogen content by controlling expression of glucose transporters, glycolytic enzymes, and pathways related to glucose homeostasis, as well as induction of mitochondrial biogenesis, fatty acid oxidation, and oxidative phosphorylation. In the context of TFEB overexpression, this effect is mostly independent of PGC-1α [152]. Several of these genes, including respiratory chain proteins of complexes II, IV, and V can be directly activated by TFEB, while others may be regulated through an indirect mechanism [152, 153, 180].

Another example of signal integration in the cell is evidenced by the connection between TFEB and the master regulators of antioxidant response, namely Nuclear Respiratory Factor 1 (NRF1) and Nuclear Factor-Erythroid-2-Related Factor 2 (*NFE2L2*, NRF2). Some of the PGC-1α-independent effects of TFEB on mitochondrial biogenesis have been ascribed to regulation of NRF1 and NRF2. TFEB can directly bind to their promoters and activate their transcription, which can lead to an upregulation of TFAM and result in increased mitochondrial volume and density [152]. In overlapping mechanisms, TFEB has also been described to participate in regulating antioxidant genes in a direct (e.g., HO1 and SOD2 [181]) or indirect way (e.g., through NRF2 [182]). In accordance with its role in balancing the cellular homeostasis, TFEB provides a contribution to the physiological reaction to stress in a concentration- and context-dependent manner. Stressors such as rotenone, CCCP, or chloramine T may trigger an MCOLN1-mediated release of Ca^2+^ from lysosomes followed by activation of calcineurin and TFEB [183]. These pathways still don’t exhaustively explain the effect of TFEB on mitochondrial biogenesis. A recent genetic screen in *Drosophila* identified TSG101 (tumor susceptibility gene 101) as regulator of mitochondrial number and size in fly neuronal axons. Mitochondrial biogenesis in neuronal axons in a *TSG101* mutant background was independent of PGC-1ɑ, NRF2, and mTOR but required TFEB and the mitochondrial fission–fusion machinery [184].

From these studies it becomes clear that TFEB, and possibly also other MiT members, play a central role in integrating mitochondrial function and metabolic homeostasis and reaction to stress. A fundamental role of TFEB in mitochondrial quality control therefore becomes obvious, and an increasing body of evidence suggests that TFEB overexpression leads to enhanced clearance of damaged mitochondria. A recent review systematically outlined this link [185]. Through the description of a functional link between PGC-1α and TFEB transcriptional activity in an animal model of HD, it became clear that MiT members play a role in promoting proteostasis, bioenergetics and mitochondrial quality control in neurodegeneration [154]. Also in iPSC-derived neurons and in a Parkin p.Q311X mouse model of PD, the PGC-1α-TFEB signaling axis was impaired, impacting the mitochondrial quality control in a PARIS-mediated manner. Inhibition of mTOR with Rapamycin or TFEB induction restored PGC-1α-TFEB signaling and abrogated impaired mitochondrial quality control and neurodegenerative features [186]. This mechanism can be linked to the action of TFEB on autophagy gene induction. One such candidate gene central to autophagy is *SQSTM1* (p62), which is upregulated upon mitophagy induction with CCCP treatment at the mRNA and protein levels [187]. TFEB and NRF2 nuclear translocation both contribute to this effect and thereby promote lysosomal biogenesis and enhance the ability of the cells to perform mitophagy [187]. Additional autophagic proteins, such as lysosomal hydrolases are also induced by TFEB nuclear translocation upon oligomycin- and antimycin-A- induced mitophagy. This way, TFEB facilitates mitochondrial clearance due to a Parkin and PINK1-dependent mechanism [150]. Chemical activation of TFEB promoted the recruitment of autophagosomes to mitochondria under physiological conditions and engaged PINK1 and Parkin to mitochondria to potentiate mitophagy under mitochondrial stress [188].

PINK1/Parkin-independent mitophagy pathways are controlled by multiple factors, including the two LC3-interacting region (LIR)-containing receptors BCL2 interacting protein 3 (BNIP3) and BCL2 interacting protein 3 Like (BNIP3L/NIX). BNIP3 and NIX can induce mitophagy upon phosphorylation and LC3 binding under hypoxic conditions and in response to high-fat diet induced lipotoxicity [189–191]. BNIP3 and TFEB are mutually regulated in a Beclin-1 and PGC-1α-dependent feedback loop that integrates mitochondrial autophagy and biogenesis [192]. Recently, two additional proteins containing lipid interacting domains, namely Cyclin G-associated kinase (GAK) and Protein Kinase C Delta (PRKCD) were identified as regulators of Parkin-independent mitophagy in vivo [193]. *GAK*, a risk gene for PD [15], directly interacts with LRRK2 [194] and controls lysosomal dynamics during autophagy [195]. Inhibition of GAK in cell lines led to nuclear localization of TFEB and increased lysosomal biogenesis, without affecting nonselective autophagy or Parkin-dependent mitophagy [193]. On the other hand, members of the PKC family of kinases have been shown to indirectly activate TFEB to control lysosomal biogenesis in an mTORC1-independent manner [196]. These studies all evidence a role of MiT members in multiple mitophagy pathways.

Conditions of mitochondrial dysfunction, such as inhibition of mitochondrial translation, combined with impaired mitochondrial dynamics, activated TFEB also in in vivo models, such as *C. elegans* to increase the lifespan of worms [197]. Proof of a physiological role of TFEB in mitochondrial homeostasis further came from unrelated in vivo models, such as the investigation of brown adipose tissue whitening in mice, for which TFEB was identified as driver through its action on the mitochondrial degradation machinery [198].

Efficient mitochondrial degradation and appropriate maintenance of mitochondrial quality control also involves fine-tuning of mitochondrial dynamics, namely mitochondrial fission and fusion. TFEB may be involved in regulating mitochondrial dynamics through regulation of expression of key mitochondrial dynamics genes [199]. The equilibrium between mitochondrial fission and fusion is regulated in concert with TFEB activation through a Drp1-mTOR-dependent mechanism [185, 200]. A summary of the functional effects of MiT transcription factors on mitochondrial biology is outlined in Fig. 1.

### Role of TFEB in neurodegenerative diseases

The role of MiT transcription factors in modulating cellular homeostasis has made them a target of interest for many pathological conditions with a metabolic background, such as lysosomal storage disorders [148, 201], cardiovascular disease [202], and NDD. Not long after the description of TFEB as central protein in a gene network regulating lysosomal biogenesis and function, it was shown that enhancing lysosomal biogenesis in cellular and animal models of PD can increase autophagolysosomal clearance and attenuate cell death. In an MPTP-driven mouse model that mimics several aspects of PD, such as nigrostriatal dopaminergic cell loss and mitochondrial dysfunction, dopaminergic neuron death is preceded by an accumulation of autophagosomes and decrease in lysosomes. Genetic induction of TFEB transcription or pharmacological enhancement of TFEB activation through mTOR inhibition with rapamycin were sufficient to enhance lysosomal biogenesis and autophagic clearance to protect from cell death [203]. TFEB overexpression prevented neuronal death and restored neuronal function in an MPTP-treated mouse model through the MAPK1/3—AKT—4EBP1—S6K1 cascade to enhance protein synthesis [204]. The translational importance of such findings was emphasized by the fact that human PD brains showed accumulations of autophagosomes [203]. Such in vivo dysfunction of the lysosomal compartment in PD brains could be due to defects in TFEB function, as shown in a 6-hydroxydopamine (6-OHDA) rat model of PD [205]. Neuroblastoma SH-SY5Y cells also showed impairment of autophagic flux and decreased lysosomal number and function upon 6-OHDA treatment, which could be ameliorated by TFEB overexpression [205]. In similar in vitro and in vivo models induced by 6-OHDA and ascorbic acid (6-OHDA/AA), it was shown that the toxic agents induced oxidative stress, which then activated a cascade involving Ca^2+^ release through the MCOLN1 channel and resulting in calcineurin-dependent activation of TFEB to promote autophagy. TFEB overexpression or pharmacological enhancement rescued SH-SY5Y cells, iPSC-derived dopaminergic neurons, and mouse nigral dopaminergic neurons from oxidative stress-induced cell death in an autophagy-dependent manner [206, 207].

Cells may upregulate lysosomal genes via TFEB induction as a compensatory response against oxidative stress in vitro and in vivo [208]. Rotenone, a respiratory chain complex I inhibitor, has long been used to establish animal models that recapitulate degeneration of the nigrostriatal dopamine system and reproduce key pathological features of clinical PD [209]. A zebrafish model of PD, created by *Pink1* deficiency and rotenone as environmental stressor, helped to identify molecules (e.g., trifluoperazines) that rescued *Pink1* deficiency by activating autophagy through TFEB activation [210]. PGC-1α was also shown to play a role in attenuation of oxidative stress, partially in a TFEB-dependent manner [154].

A direct genetic link between lysosomal defects and PD risk is given by pathogenic variants of the *GBA1* gene. In a *Drosophila* model of GD with *GBA1* deficiency, a block in autophagy flux was demonstrated together with downregulation of mTOR signaling and compensatory increase in *Mitf* gene expression. Further pharmacological mTOR inhibition ameliorated the fly phenotype, implicating a role for mTOR in this *GBA1*-deficiency model [211]. A biological difference between homozygous and heterozygous *GBA1*-deficiency was shown in a study of 3D neurosphere models, in which only heterozygous mutant cells were able to compensate for lysosomal deficiencies and impaired mitochondria by upregulating TFEB and PGC-1α [212]. TFEB gene expression was confirmed to be upregulated in the cortex of patients with Lewy-body disease in anti-correlation with *GBA1* expression [213]. Whether MiT proteins in this context present a compensatory mechanism to the disease or are involved in disease etiology is still an open question. However, upregulation of MiT signaling has been proposed as viable therapeutic target in another glycogen storage disease, namely Pompe disease. In this syndrome, caused by alpha-Glucosidase (GAA) deficiency, TFEB overexpression alleviated disease markers in vitro and in vivo through the induction of exocytosis of autophagolysosomes [201].

In addition, mutations in *ATP13A2* have been proposed to act by decreasing *SYT11* (*Synaptotagmin 11*), another PD risk gene, through transcriptional and post-translational mechanisms, to induce lysosomal dysfunction and impaired degradation of autophagosomes. The indirect transcriptional regulation of SYT11 was achieved through MYCBP2 (MYC Binding Protein 2)-induced ubiquitination of TSC (tuberous sclerosis complex), resulting in mTORC1 activation and decreased TFEB transcriptional activation towards SYT11. Decreased SYT11 then led to lysosomal dysfunction and impaired autophagosome degradation [214].

Likewise, an important role for TFEB-mediated exocytosis has been described for tau pathologies. Lysosomal exocytosis of truncated mutant tau species lacking the microtubule-binding repeat (MTBR) domains have been shown to undergo active secretion mediated by TFEB and the lysosomal calcium channel TRPML1 (MCOLN1). In this way, TFEB plays an essential role in the lysosomal exocytosis of truncated mutant tau species [215].

Similar results were reproduced in a rat model of α-Syn, in which TFEB was primarily found to remain localized to the cytoplasm, leading to lower lysosomal markers in diseased midbrain neurons [216]. In this in vivo model, upregulation of TFEB or treatment with mTOR inhibitors was neuroprotective, while miRNA-driven downregulation of TFEB aggravated the formation of α-Syn oligomers [216]. Diseased cells with α-Syn aggregations may enter a vicious cycle, whereby α-Syn aggregates impair the ubiquitin–proteasome system and the autophagy-lysosomal pathway and enhance disease progression. Pharmacological and genetic activation of TFEB may help to escape this spiral by promoting autophagic clearance of aggregated α-Syn [217]. An A53T α-Syn PD rat model also benefitted from TFEB overexpression in neurons to prevent neurodegeneration [218]. Aggregated α-Syn may also be transmitted from cell to cell, a mechanism that is in part dependent on iron accumulation and resulting inhibition of mTORC1-TFEB-dependent autophagosome‐lysosome fusion [219]. Recently, an indirect mechanism of TFEB activation has been shown to improve the motor ability of an α-Syn A53T transgenic mouse model. PARP1 (Poly-ADP-Ribose Polymerase 1) inhibitors, such as Veliparib, prevented neurodegeneration in vivo through TFEB activation via SIRT1-mediated downregulation of mTOR and reduced TFEB nuclear export by attenuating the TFEB-CRM1 interaction [220].

### MiT members as pharmacological targets in PD

The above-mentioned models point towards MiT members as strong candidates to ameliorate PD pathology through activation of the ALP. Direct pharmacological activation of proteins, especially transcription factors, is however a difficult task. Also, activation of MiT transcription factors should be achieved specifically in the diseased cells and at an early stage of disease, while neuronal death is still preventable. Therefore, early diagnostic tools will be necessary to achieve significant therapeutic effects with small molecules or biologicals. On the other hand, the important role of MiT transcription factors in driving several cancers precludes a straightforward possibility to use gene therapy approaches to upregulate the activity of these transcription factors. Nevertheless, several efforts are underway to identify molecules that could directly or indirectly activate these transcription factors to exert their beneficial effects in preventing or treating PD defects.

Based on the molecular mechanism of MiT protein activation, it became clear that mTORC1 constitutes an attractive therapeutic target. Due to its properties to accelerate the clearance of aggregated toxic proteins, mTOR modulators have long been proposed as potential drugs against PD [203, 221]. As in Parkin p.Q311X in vitro and in vivo models, rapamycin was able to activate autophagy also in affected brain tissue of an MPTP mouse model [186, 222]. mTOR inhibitors, such as rapamycin, but not ribosomal protein S6 kinase inhibitors, have been indicated in in vivo models as combination therapy with standard antiparkinsonian agents to alleviate motor symptoms and cognitive symptoms of PD [223, 224]. Interestingly, clinical administration of everolimus, a rapamycin analog, resulted in a significant improvement of memory and affective performance, which are side-effects of immunosuppression using calcineurin inhibitors, in patients requiring immune suppression [225]. Sirolimus and novel mTOR inhibitors are currently being tested in clinical trials in PD and multiple system atrophy (MSA) patients (clinicaltrials.gov: NCT03589976; anzctr.org.au: ACTRN12619000372189). However, the trial on MSA was stopped as no beneficial effect for disease progression was detected, while the status of the trial on PD is currently not updated. Further characterizing the dysregulation of mTOR pathway and the clinical translation of mTOR modulators in PD may offer exciting new avenues for future drug development. Yet, because mTOR signaling is essential for multiple cellular functions, a certain level of mTOR activity is necessary and long-term systemic and even neuron-specific mTOR inhibition may be of limited clinical benefit [226]. Therefore, alternative small molecules have been investigated that would exploit similar pathways but would spare canonical mTOR inhibition. Dynasore, a small molecule GPTase inhibitor targeting dynamin is such a candidate. It has been shown to repress the lysosomal localization and activity of mTORC1, which in turn enhanced the nuclear translocation of TFE3 and TFEB, leading to increased autophagic flux. Its efficacy at promoting clearance of protein aggregates was shown in an in vitro model of HD [227]. More relevant to PD, it was shown that Dynasore can increase the uptake of α-Syn protein by cultured microglia cells [228] and inhibit α-Syn protein transfer between cells in a co-culture model [229].

Trehalose is a naturally occurring sugar consisting of two molecules of glucose that is being used in food, cosmetics, and drugs. When ingested by humans, it is rapidly broken down into glucose by intestinal trehalase. In biological systems, trehalose has been shown to exert a potent pro-autophagic activity both in vitro and in vivo. As such it has been shown to be neuroprotective in a *parkin/tau* mouse model of tauopathy [230]. As a molecular chaperone, it prevented motor deficits in an MPTP mouse model by providing protection against neuroinflammation through its action against microglia, astrocytes, and endothelial cells [231]. Likewise, an AAV-overexpressing α-Syn rat model of PD benefitted from high doses of trehalose through autophagy enhancement [232]. Recently, the activity of trehalose has been described to function by inducing a rapid transient lysosomal enlargement and membrane permeabilization leading to calcineurin activation, TFEB dephosphorylation and activation, and resulting in upregulation of lysosomal and autophagy genes [233]. Combining trehalose with the mTOR inhibitor rapamycin had an additive effect on autophagy activation and led to reversal of neuronal and behavioral deficits in vivo in an MPTP-induced mouse model of PD [222]. Yet, trehalose needs to be administered intravenously to avoid intestinal breakdown, which warrants the development of trehalase-resistant analogs, such as Lentztrehalose [234].

Another oligosaccharide, 2-Hydroxypropyl-β-cyclodextrin (HPβCD), has also been shown to activate TFEB and upregulate lysosomal biogenesis and autophagy. As US Food and Drug Administration-approved drug delivery vehicle, it is deemed safe and was recently shown to exert pharmacological properties by itself in models for cholesterol storage disorders, such as NPC, which may be due to its activation of TFEB [235, 236].

The quest for TFEB activators has also led to the identification of curcumin and its derivatives as promising small molecule leads. Curcumin is a bright yellow chemical produced by species of the *Curcuma longa* plant that has been ascribed anti-inflammatory properties. Curcumin ameliorates oxidative stress on intestinal epithelial cells in an AMPK-, TFEB-, and Parkin-dependent mechanism [237]. The orally available curcumin derivative C1 was described as mTOR-independent activator of TFEB that promoted nuclear translocation and activation by direct binding of TFEB and thereby enhancing autophagy and lysosomal biogenesis in cell and animal models [238]. The same compound was also used in an AD mouse model to increase autophagy and lysosomal activity and improve synaptic and cognitive function [239]. Another Curcumin analog, C4, was shown to activate TFEB through AKT-mTORC1 inhibition and resulted in α-Syn degradation and cytotoxicity protection in a PD cell model [240]. Related evidence showed that curcumin was effective in preventing mitochondrial hexokinase 1 (HK1) release and ROS protection following α-Syn fibrillation product injury in vitro [241].

A beneficial effect of anti-inflammatory drugs on PD phenotypes through the MiT pathway has recently gained some evidence in in vitro models. Neuroinflammation is increasingly being recognized as important player in PD and as a potential mechanistic link between defective mitochondrial quality control and PD pathogenesis [242]. Celecoxib, a clinically approved selective cyclooxygenase 2 inhibitor, had the potential to rescue PD cell models challenged with 6-OHDA or paraquat [243]. The non-selective phosphodiesterase inhibitor and anti-inflammatory drug Ibudilast was shown to act as an autophagy enhancer through mTORC1 inhibition and TFEB activation [244]. While subtoxic levels of tumor necrosis factor alpha (TNFα), a pro-inflammatory cytokine involved in neurodegeneration in PD, led to impaired autophagic flux, mTOR inhibition with PP242 increased TFEB activation and partially rescued the TNFα effect [245].

Ambroxol, a mucolytic agent used as active ingredient of cough syrup, acts as a small molecule chaperone for GCase and improves lysosomal function and GCase activity in vitro and in vivo [246]. The drug increased brain GCase enzyme activity or GCase protein levels in *Drosophila* [247], rodents [248], non-human primates and PD patients, both with and without *GBA1* gene mutations [249]. It has been shown that enhancement of GCase activity through small molecules (i.e., 758) can reduce the levels of soluble α-Syn [250, 251]. Accordingly, ambroxol decreased α-Syn or phospho-α-Syn levels in in vitro and in vivo models of PD [41, 248, 252, 253]. It is further being tested in clinical trials for its safety, tolerability and efficacy against PD and PD dementia (clinicaltrials.gov: NCT02941822 [249]; NCT02914366 [254]). Notably, a trial on 18 participants suggested that ambroxol therapy was safe and well tolerated and was able to reduce the mean (SD) scores on part 3 of the Movement Disorders Society Unified Parkinson Disease Rating Scale [249]. Mechanistically, ambroxol has been shown, among other potential mechanisms, to increase and activate TFEB and stimulate lysosomal exocytosis [246, 248]. Additional small molecules shown to potentially act through TFEB activation are PARP1 inhibitors, such as Veliparib in an α-Syn mouse model [220] or trifluoperazine, an inhibitor of calmodulin and dopamine D2 receptor, in *Pink1* deficiency and rotenone-induced zebrafish models with PD-like symptoms [210].

A summary of drug candidates activating MiT members that have been investigated in in vitro and in vivo models, and some of which have reached different stages of clinical trials, are presented in Table 3 and Fig. 2.

**Table 3:** Summary of the drug development status for the activation of MiT members

Compound	Mechanism of action	Disease / symptoms	Development stage	References / Clinical Trial
Rapamycin	Inhibitor of mTOR; autophagy activation; mitochondrial quality; control MiT activation	PD	Preclinical (in vitro and in vivo)	[186, 222]
Everolimus	Inhibitor of mTOR; immunosuppressant; inhibition of T-cell and B-cell proliferation	Immuno-suppression in heart transplant recipients	Clinical (pilot study)	[225]
Sirolimus and RTB101 (alone or in combination)	Inhibitors of mTOR; autophagy activation	PD; MSA	Clinical trial	anzctr.org.au (trial ID: ACTRN12619000372189), phase 1/2, trial information not updated; clinicaltrials.gov (trial ID: NCT03589976), phase 2, trial stopped
Dynasore	GTPase inhibitor targeting dynamin; TFE3 and TFEB activation	HD; PD	Preclinical (in vitro)	[227–229]
Trehalose	Naturally occurring sugar; TFEB activation	Tauopathy; PD	Preclinical (in vitro and in vivo)	[222, 230–232]
2-Hydroxypropyl-β-cyclodextrin (HPβCD)	Drug delivery vehicle; TFEB activation	cholesterol storage disorder (NPC)	Preclinical (in vitro); clinical trial	[235, 236]; clinicaltrials.gov (trial ID: NCT03893071), phase 1/2; clinicaltrials.gov (trial ID: NCT03893071), phase 2/3
Curcumin and its derivatives C1, C4	mTOR-independent TFEB activators	AD; PD	Preclinical (in vitro and in vivo)	[237, 239–241]
Celecoxib	Cyclooxygenase 2 inhibitor; TFEB activation	PD	Preclinical (in vitro)	[243]
Ibudilast	Anti-inflammatory phosphodiesterase inhibitor; TFEB activation		Preclinical (in vitro)	[244]
PP242	mTORC1 inhibitor; TFEB activation	PD	Preclinical (in vitro)	[245]
Ambroxol	GCase chaperone; TFEB activation	PD	Preclinical (in vivo); clinical trial	[246, 248, 249]; clinicaltrials.gov (trial ID: NCT02941822), phase 2 completed; clinicaltrials.gov (trial ID: NCT02914366), phase 2
Veliparib	PARP1 inhibitor; TFEB activation	PD	Preclinical (in vivo)	[220]
Trifluoperazine	Calmodulin and Dopamine receptor blockade; TFEB activation	PD	Preclinical (in vivo)	[210]

Pharmacological compounds with a described direct or indirect action on the MiT pathway is provided. For each compound a proposed mechanism of action is listed, together with the disease for which the compound has been tested in connection with MiT pathway activation. The preclinical or clinical development stage of the drugs are shown with references describing the studies and relevant clinical trials

### Genetic evidence for involvement of MiT target gene as disease modifiers in PD

Further hints towards a role of the MiT transcription factors and lysosomal biogenesis pathway in PD come from genetics signals that involve several MiT target genes as PD risk genes and disease modifiers. One such gene is *GPNMB* (osteoactivin), encoding transmembrane glycoprotein non-metastatic melanoma protein B, which is induced upon MiT activation and is mainly expressed in melanocytes, osteoclasts, dendritic cells and overexpressed in various cancers and lysosomal storage disorders. *GPNMB* is a direct transcriptional target of TFE3 [255, 256] and MITF [257] and may be involved in cell adhesion, migration, proliferation, and differentiation [258]. Recent large-scale genome-wide association studies detected genetic signals close to or on the *GPNMB* gene locus, associated with age of onset of PD and PD risk [259, 260]. Similar genetic links between *GPNMB* and PD risk were also shown when taking into account eQTL (expression quantitative trait loci) data in blood or brain tissues [261, 262]. Likewise, mendelian randomization approaches on gene expression datasets aimed at investigating druggable targets for PD, identified *GPNMB* among other genes as potentially targetable protein against PD [263, 264]. In line with these data, *GPNMB* has been shown to be selectively elevated in the *SNpc* of PD patients, in addition to other amyloid related NDDs [265, 266]. An additional link between our current understanding of PD etiology and a potential role of *GPNMB* may come from recent studies that involved the protein in inflammation and neuroinflammation, even though its exact role in inflammation is not well established so far (reviewed by Saade et al., 2021 [267]). These data allow for the speculation that MiT downstream genes may provide a link between various aspects of PD pathology, spanning from metabolic to autophagic and inflammatory responses.

## Conclusions

PD is a NDD that is characterized by pathological protein misfolding and aggregation as well as organellar dysfunction leading to neuronal cell death, which causes motor-and non-motor symptoms. Mitochondrial homeostasis and quality control have historically been recognized as crucial contributors to PD pathogenesis, and several aspects of mitochondrial biology are impaired in PD patients and models. In addition, defects of macroautophagy and the ALP have been observed in cell and animal models of PD as well as PD patients’ brains, where constitutive autophagy is indispensable for adaptation to stress and energy deficiency. Various mechanisms are involved in the interplay between PD-related mitochondrial defects, lysosomal dysfunctions, and protein aggregate formations. The specific disease cascade in PD patients may differ based on which genetic and environmental factors act as main triggers. As outlined in this review, the functions of various organellar compartments are tightly linked and influence each other. Connections between these organelles are constituted among others by mitophagy, metabolite homeostasis, organellar dynamics, including exo- and endocytosis, and cellular signaling cascades, including Ca^2+^ signaling, mTOR signaling and the activation of transcription factors. Members of the MiT family of transcription factors are central to this connection as they are regulated by several of the nodes linking mitochondria and lysosome functions. MiT transcription factors can trigger a downstream transcriptional program aimed at responding to nutrient stress through the regulation of lysosomal biogenesis and autophagy. This property has been found as promising therapeutic target against NDDs, including PD. Several genetic and pharmacological approaches have shown encouraging results in in vitro and in vivo models whereby the activation of MiT transcription factors can ameliorate PD-related phenotypes. However, direct and specific activations of transcription factors is difficult to achieve pharmacologically. Therefore, it will be necessary to gain a deeper understanding of how MiT transcription factors are regulated and activated in various cell types. This may lead to the recognition of druggable proteins that can influence MiT activation and help to develop pharmacologically relevant molecules that would increase lysosomal biogenesis and boost degradation pathways in the brain of affected individuals. Such treatment strategies would best be applied before debilitating effects of the disease are being experienced. This, however, will require parallel efforts for the development of early diagnostic methods that would help to recognize biological malfunctions before the damage to cells is too extensive and clinical symptoms appear. Only if disease-altering treatment options are applied before significant brain regions are impacted by the disease, it will be possible to achieve truly meaningful effects.

Overall, more work will be needed to bridge basic knowledge of the main pathways affecting PD with their potential clinical applications, but recent developments in the field have uncovered promising pathways, including the here described approaches, that may provide disease-altering therapies against PD.

## Data Availability

Not applicable.

## Abbreviations

AD: Alzheimer’s disease
ALP: Autophagy-lysosomal pathway
AMPK: AMP-dependent protein kinase
α-Syn: Alpha-Synuclein
CRM1: Exportin 1
iPSC: Induced pluripotent stem cell
L-Dopa: Levodopa
ER: Endoplasmic reticulum
FLCN: Folliculin
FNIP1/FNIP2: FLCN interacting proteins
GCase: Glucocerebrosidase
GD: Gaucher disease
HD: Huntington’s disease
HPβCD: 2-Hydroxypropyl-β-cyclodextrin
LRRK2: Leucine-Rich Repeat Kinase 2
LSDs: Lysosomal storage disorders
MCOLN1: Mucolipin1
MiT: Microphthalmia-associated transcription factor family
MITF: Microphthalmia-Associated Transcription Factor
MPP +: 1-Methyl-4-phenylpyridinium
MPTP: 1-Methyl-4-phenyl-1,2,3,6-tetrahydropyridine
mTORC1: Mammalian target of rapamycin complex 1
MSA: Multiple system atrophy
NDD: Neurodegenerative disorder
NPC: Niemann-Pick type C
NRF1: Nuclear Respiratory Factor 1
NRF2, NFE2L2: Nuclear Factor-Erythroid-2-Related Factor 2
6-OHDA: 6-Hydroxydopamine
PARP1: Poly(ADP-Ribose)-Polymerase 1
PD: Parkinson’s disease
iPD: Idiopathic Parkinson’s disease
PGC-1α: Peroxisome proliferator-activated receptor gamma coactivator-1alpha
PI3K/Akt: Phosphoinositide 3-kinases/ Protein kinase B (PKB), also known as Akt
PINK1: PTEN Induced Kinase 1
RAG: Ras-related small GTPases
RCC: Renal cell carcinoma
ROS: Reactive oxygen species
SIRT1: Sirtuin-1
SNpc: Substantia nigra pars compacta
SYT11: Synaptotagmin 11
TFAM: Mitochondrial transcription factor A
TNFα: Tumor necrosis factor alpha
TSC: Tuberous sclerosis complex
TSG101: Tumor susceptibility gene 101
VPS35: Vacuolar protein sorting-associated protein
mtDNA: Mitochondrial genome
eQTL: Expression quantitative trait loci
